# Regulatory mechanism of O‐linked N‐acetylglucosamine protein modification on autophagy in cancer

**DOI:** 10.1002/ctm2.70596

**Published:** 2026-01-15

**Authors:** Yizhan Li, Ling Gao, Shaoming Li, Jingjing Zheng, Keqian Zhi, Wenhao Ren

**Affiliations:** ^1^ Department of Oral and Maxillofacial Reconstruction The Affiliated Hospital of Qingdao University Qingdao China; ^2^ School of Stomatology Qingdao University Qingdao China; ^3^ Key Lab of Oral Clinical Medicine The Affiliated Hospital of Qingdao University Qingdao China; ^4^ Department of Stomatology The Affiliated Hospital of Qingdao University Qingdao Shandong China

**Keywords:** autophagy, cancer, metabolic reprogramming, O‐GlcNAcylation

## Abstract

**Background:**

O‐linked N‐acetylglucosamine protein modification (O‐GlcNAcylation) is a dynamic, nutrient‐sensitive post‐translational modification frequently upregulated in cancers. Autophagy, a lysosome‐dependent recycling pathway, plays a context‐dependent dual role in tumorigenesis and therapy resistance. Emerging evidence reveals intricate crosstalk between these two processes, positioning the O‐GlcNAcylation‐autophagy axis as a critical regulator of cancer cell adaptation.

**Main Topics:**

This review systematically delineates the multidimensional mechanisms by which O‐GlcNAcylation regulates distinct stages of autophagy initiation, maturation, and fusion across various cancer types. We detail how O‐GlcNAcylation targets core autophagy machinery, including the ULK1 complex, LC3 lipidation system, and SNARE fusion proteins, and modulates key signaling hubs like mTOR and AMPK. Furthermore, we integrate this molecular regulation with the stage‐specific pro‐tumor or tumor‐suppressive functions of autophagy, highlighting how O‐GlcNAcylation remodels autophagic flux to promote metabolic reprogramming, stress survival, and therapeutic resistance.

**Conclusions:**

The O‐GlcNAcylation‐autophagy axis represents a promising therapeutic target. Combining small‐molecule inhibitors of O‐GlcNAc cycling enzymes (OGT/OGA) with autophagy modulators offers a novel strategy to overcome tumor drug resistance. Future research must address the heterogeneity of this regulatory network across cancer types and developmental stages to advance precision oncology interventions.

**Keypoints:**

- O‐GlcNAcylation serves as a nutrient and stress sensor that dynamically regulates autophagy at multiple stages in cancer cells.
- It fine‐tunes autophagy initiation, maturation and fusion by modifying key proteins such as ULK1, ATG4B and SNAP‐29.
- Context‐dependent O‐GlcNAcylation promotes tumour adaptation and therapy resistance via autophagy remodelling.
- Targeting the O‐GlcNAc–autophagy axis offers a promising strategy to overcome cancer drug resistance.

## Graphical abstract

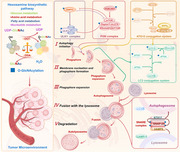

## 1 INTRODUCTION

O‐linked N‐acetylglucosamine protein modification (O‐GlcNAcylation) is a dynamic modification wherein O‐GlcNAc transferase (OGT) catalyses the addition of N‐acetylglucosamine (GlcNAc) from uridine diphosphate‐N‐acetylglucosamine (UDP‐GlcNAc) to serine/threonine residues; the reverse reaction is performed by O‐GlcNAcase (OGA).^1^ This modification responds rapidly to environmental cues, thereby modulating protein activity and stability. The hexosamine biosynthetic pathway (HBP) serves as a major nutrient‐sensing route, converting glucose, glutamine, acetyl‐CoA and uridine triphosphate (UTP) into UDP‐GlcNAc through a series of enzymatic steps.^2^ HBP activity is regulated by various signalling molecules, including the mechanistic target of rapamycin (mTOR) signalling pathway in response to environmental signals, AMP‐activated protein kinase (AMPK) and stress‐regulated transcription factors, which adjust HBP flux according to cellular nutrient and stress conditions.^3^, ^4^, ^5^ Dysregulated O‑GlcNAcylation is implicated in various diseases, including cancer, diabetes and neurodegenerative disorders.^6^ In tumours, O‐GlcNAcylation is frequently up‐regulated and promotes cell proliferation and survival by directly modifying key transcription factors (e.g., forkhead box protein M1 (FOXM1), nuclear factor kappa‐B (NF‐κB)) or signalling effectors (e.g., Yes‐associated protein (YAP)).^7^, ^8^

Autophagy is a lysosome‐dependent recycling process that sustains cellular homeostasis. It proceeds through several stages: induction and phagophore formation, autophagosome maturation and fusion with lysosomes to form autolysosomes.^9^ Based on the delivery route of cargo to lysosomes, autophagy is classified into macroautophagy, microautophagy and chaperone‐mediated autophagy; this review focuses on macroautophagy, the best‑characterised form.^10^, ^11^ Autophagy is regulated by a network of signalling pathways and autophagy‐related genes (ATG) proteins.^12^ In cancer, autophagy exerts context‑dependent ‘double‑edged sword’ effects: it can suppress early tumourigenesis by removing damaged organelles and oncogenic proteins, yet in established tumours it may sustain cell survival under stress and contribute to therapy resistance.^13^ Therefore, understanding the precise role of autophagy in specific tumour contexts is vital for developing effective treatments.

O‐GlcNAcylation regulates autophagy through diverse mechanisms, including direct modification of autophagy‐related proteins, modulation of upstream signalling pathways, control of autophagosome–lysosome fusion and responsiveness to metabolic changes. Many proteins involved in different stages of autophagy are known O‐GlcNAcylation targets.^14^, ^15^ While previous reviews have discussed O‑GlcNAc biology or autophagy separately, this article uniquely integrates cancer‑specific mechanisms, with a focus on stage‑resolved control of autophagy (initiation, maturation, fusion) by O‑GlcNAcylation and its direct impact on therapeutic resistance. We emphasise the coupling of nutrient‑sensing pathways with autophagic flux, providing a coherent framework for understanding how tumour cells exploit the O‑GlcNAcylation–autophagy axis for metabolic adaptation. This review systematically examines how O‑GlcNAcylation, OGT and OGA regulate distinct phases of autophagy in cancer, and how reprogramming autophagic flux through this post‐translational modification (PTM) influences metabolic plasticity and therapy resistance. These insights advance our understanding of the O‑GlcNAcylation–autophagy axis and provide a rationale for targeting it with combination therapies, such as OGT inhibitors together with autophagy modulators.

## 2 O‐GLCNACYLATION: NUTRIENT AND STRESS SENSORS

**FIGURE 1:**
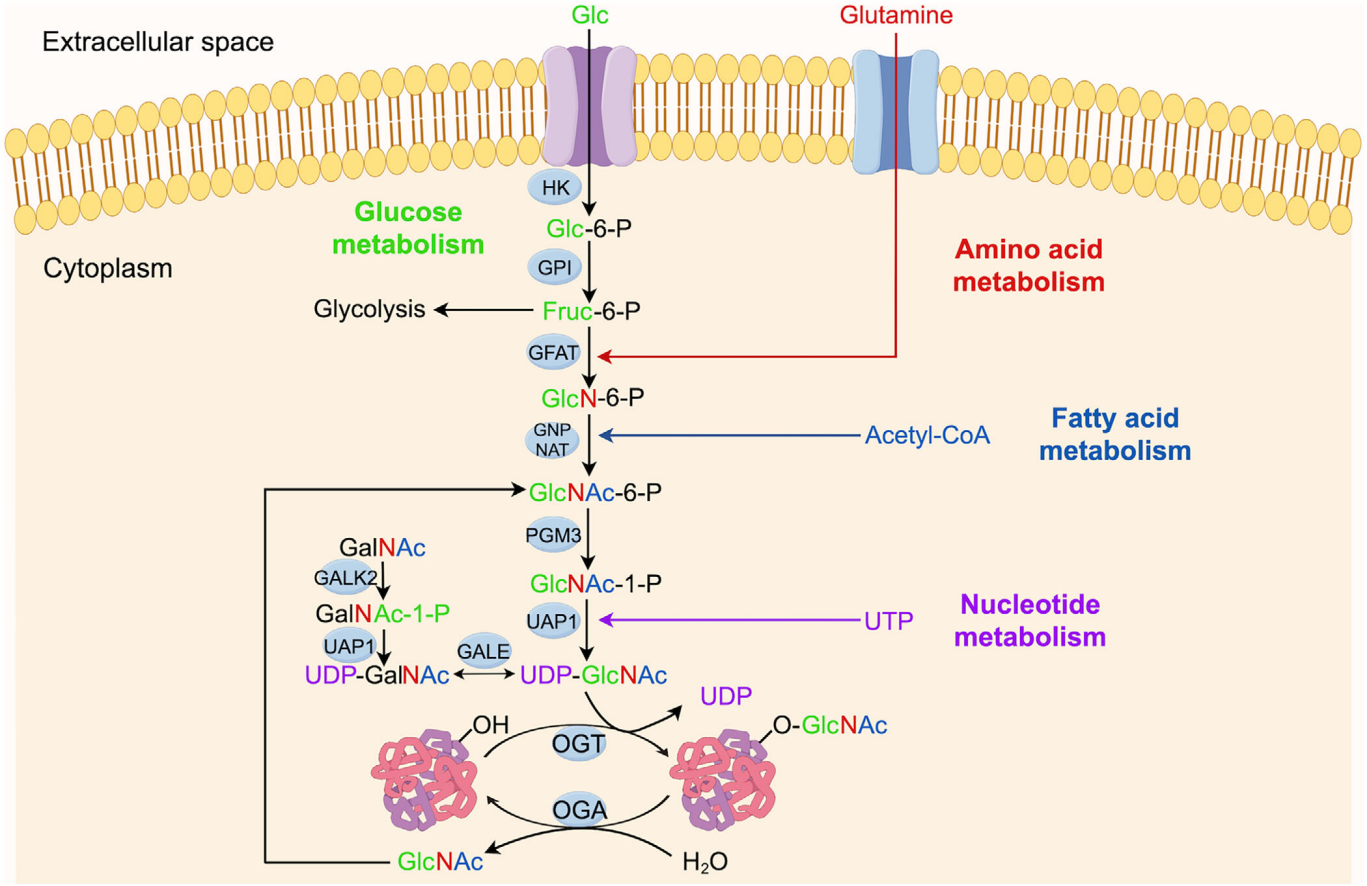
O‑GlcNAcylation serves as a central metabolic integrator and cellular nutrient sensor. O‑GlcNAcylation is a dynamic post‑translational modification whose donor substrate, UDP‑GlcNAc, is synthesised via the hexosamine biosynthesis pathway (HBP). This figure illustrates how the HBP integrates and senses key precursors derived from glucose, amino acid (glutamine), fatty acid and nucleotide metabolism, thereby establishing O‑GlcNAcylation as a critical intracellular ‘nutrient and stress sensor’. Extracellular glucose (Glc) enters the cytosol via glucose transporters (GLUTs), is phosphorylated by hexokinase (HK) to glucose‑6‑phosphate (Glc‑6‑P) and then converted by phosphoglucose isomerase (GPI) to fructose‑6‑phosphate (Fruc‑6‑P). The rate‑limiting enzyme glutamine: fructose‑6‑phosphate amidotransferase (GFAT) uses the amino group from glutamine to convert Fruc‑6‑P into glucosamine‑6‑phosphate (GlcN‑6‑P). Acetyl‑CoA (derived from fatty acid metabolism) is then utilised by N‑acetylglucosamine‑6‑phosphate synthase (GNPNAT) to generate N‑acetylglucosamine‑6‑phosphate (GlcNAc‑6‑P). GlcNAc‑6‑P is converted to GlcNAc‑1‑P by phosphoacetylglucosamine mutase (PGM3) and finally, with uridine triphosphate (UTP, derived from nucleotide metabolism), is catalysed by UDP‑N‑acetylhexosamine pyrophosphorylase (UAP1) to yield UDP‑GlcNAc, the direct donor for O‑GlcNAcylation. The GlcNAc moiety of UDP‑GlcNAc is transferred to serine/threonine residues of target proteins by O‑GlcNAc transferase (OGT). This modification can be hydrolytically removed by O‑GlcNAcase (OGA), maintaining the dynamic equilibrium of protein O‑GlcNAcylation in response to nutrient and metabolic signals.

O‐GlcNAcylation, a widespread PTM in eukaryotes, has been identified in more than 4000 proteins.^16^ Its modification substrate, UDP‐GlcNAc, is mainly derived from the HBP, a branch of glucose metabolism whereby 95% of glucose ingested by the cell is metabolised through glycolysis for energy supply. The remaining 3–5% enter the HBP.^17^ Subsequently, metabolic precursors such as glucose, glutamine, acetyl coenzyme A and UTP are catalysed by key enzymes, such as glutamine fructose‐6‐phosphate aminotransferase (GFAT), to generate UDP‐GlcNAc ultimately.^18^ Because the HBP integrates inputs from glucose, amino acid, lipid and nucleotide metabolism, O‐GlcNAcylation is considered an intracellular ‘nutrient sensor’ (Figure 1). As a nutrient sensor, O‐GlcNAcylation dynamically monitors changes in nutrient levels and adjusts target protein activity, stability and interactions, thereby coordinating cell‐cycle progression, mitochondrial function and overall metabolic homeostasis.

Notably, O‐GlcNAcylation also functions as a central mediator of cellular stress responses.^1^ Under oxidative stress, it enhances reactive oxygen species (ROS) scavenging by modifying antioxidant enzymes such as catalase and superoxide dismutase.^19^ In response to DNA damage, O‐GlcNAcylation influences cell fate decisions by regulating cell‐cycle proteins.^20^ The crosstalk between O‐GlcNAcylation and phosphorylation constitutes another key regulatory layer: the two modifications often compete for the same or adjacent serine/threonine residues, yet they can also act synergistically, with O‑GlcNAcylation sometimes priming subsequent phosphorylation or stabilising proteins to amplify signalling outputs.^21^, ^22^, ^23^ This complex crosstalk fine‐tunes the activity of key signalling pathways, such as NF‐κB, p53 and so on.^24^, ^25^ Moreover, O‐GlcNAcylation can modulate protein kinase A by affecting its subcellular localisation and kinase activity, thereby influencing downstream phosphorylation events.^26^, ^27^ As a stress sensor, O‐GlcNAcylation thus serves as a molecular hub linking stress perception to adaptive responses, and its dysfunction contributes to disease mechanisms such as tumour metabolic reprogramming.

The enzymes responsible for this dynamic modification are OGT and OGA, which act as the ‘writer’ and ‘eraser’ of O‐GlcNAcylation, respectively^1^ (Figure 1). OGT exists in three distinct splice isoforms within cells: ncOGT, mOGT and sOGT. The ncOGT contains 13.5 tetrapeptide repeat sequences (TPRs), which mainly regulate chromatin remodelling in the nucleus. mOGT contains 9 TPRs, which regulate mitochondrial function through N‐terminal mitochondrial localisation signals. sOGT contains 2.5 TPRs, which are mainly involved in the cytoplasmic stress response. All isoforms share the C‐terminal catalytic domain and a phosphatidylinositol‐binding module, with TPR number determining substrate selectivity and localisation.^1^, ^28^, ^29^ The MGEA5 gene encodes OGA in two splice isoforms: sOGA and ncOGA. sOGA contains only the glycoside hydrolase structural domain and the OGT‐binding region. In contrast, ncOGA additionally possesses a C‐terminal pseudohistory acetyltransferase (HAT) structural domain, which may affect gene silencing through epistatic regulation.^28^, ^30^, ^31^ Although OGT and OGA are widely distributed in the nucleoplasmic bi‐region, OGT is enriched in the nucleus through nuclear localisation signalling, whereas OGA relies on anchoring proteins to localise in the cytoplasm. Under physiological conditions, O‐GlcNAcylation levels are maintained by a balanced ‘write–erase’ cycle; in pathological states such as cancer, overexpression of OGT or loss of OGA activity disrupts this homeostasis, driving malignancy through reprogramming of metabolic, epigenetic and signalling networks.

## 3 O‐GLCNACYLATION IN ONCOLOGY

O‐GlcNAcylation acts as a dynamic enzymatic modification reaction, and the efficiency of its occurrence is triply regulated by substrate abundance (UDP‐GlcNAc) and catalase (OGT/OGA) expression and activity. Tumour cells often augment HBP flux via metabolic reprogramming. For instance, oncogenic activation (e.g., by kirsten rat sarcoma viral oncogene homolog (KRAS)) elevates the transcription and activity of the rate‐limiting enzyme GFAT, boosting UDP‑GlcNAc production. Concurrently, increased OGT expression or suppressed OGA activity further amplifies global O‑GlcNAcylation levels.^32^ Consequently, tumour tissues generally exhibit higher O‑GlcNAcylation than normal counterparts, as documented in breast, liver, colon and lung cancers. This elevation frequently associates with advanced stage, metastasis and poor prognosis, supporting its potential as both a prognostic marker and a therapeutic target.^33^, ^34^

Dysregulated O‐GlcNAcylation is closely linked to malignant phenotypes.^35^ Functioning as a nutrient and stress sensor, it modulates tumour progression by modifying key signalling proteins. In the NF‐κB pathway, for example, O‑GlcNAcylation of NF‑κB at threonine residues impedes its interaction with I‑κB, leading to sustained NF‑κB activation, transcription of pro‑proliferative genes and accelerated malignant transformation.^36^ Furthermore, O‑GlcNAcylation of the tumour suppressor forkhead box O3 (FOXO3) at Ser284 promotes murine double minute 2 (MDM2)‑mediated p53 degradation, which down‐regulates p21, releases cell‑cycle arrest and facilitates tumour growth.^37^

Beyond its direct effects, O‐GlcNAcylation engages in extensive crosstalk with other PTMs, forming a complex regulatory code that fine‐tunes protein function in tumours. As previously mentioned, the interplay with phosphorylation is a cornerstone of this crosstalk. Furthermore, O‐GlcNAcylation can directly compete with or regulate ubiquitination, thereby influencing protein stability.^38^ For instance, O‐GlcNAcylation of the m^6^A reader protein YTH domain‐containing family protein 2 (YTHDF2) at Ser263 inhibits its ubiquitin‐dependent degradation, leading to its stabilisation and enhanced oncogenic activity.^39^ Conversely, O‐GlcNAcylation of the iron metabolism regulator transferrin receptor 1 (TFRC) at Ser687 promotes its ubiquitination and degradation, suggesting a context‐dependent role.^40^ This intricate PTM crosstalk underscores the multifaceted role of O‐GlcNAcylation in orchestrating the oncogenic network.

O‐GlcNAcylation also plays a pivotal role in tumour metabolic reprogramming. Tumour cells produce energy primarily through glycolysis rather than oxidative phosphorylation, even under oxygen‐sufficient conditions, a phenomenon termed the ‘Warburg effect’.^41^ Glycolysis can generate the ATP required for cell proliferation and mitigate cell damage induced by ROS produced during oxidative phosphorylation. Tumour cells achieve metabolic remodelling through the Warburg effect, in which O‐GlcNAcylation plays a multidimensional regulatory role. O‐GlcNAcylation inhibits the activity of phosphofructokinase 1 by modifying it, which induces glucose to shift to the pentose phosphate pathway (PPP), increases the production of nucleotide precursors, lipids and glyceraldehyde‐3‐phosphate dehydrogenase (GAPDH)f, and thereby supporting tumour biosynthetic requirements.^42^ In addition, O‐GlcNAcylation activates phosphoglycerate kinase 1 through glycosylation, promoting its mitochondrial translocation and enhancing the efficiency of the glycolysis–oxidative phosphorylation coupling, thereby contributing to the ‘Warburg effect’ in tumours.^43^

Beyond glycolysis, O‑GlcNAcylation acts as a master coordinator that integrates multiple metabolic pathways to fuel tumour growth. It regulates glutamine metabolism by modulating the stability and activity of key enzymes and transcription factors.^44^ In lipid metabolism, O‐GlcNAcylation of ATP‐citrate lyase promotes acetyl‐CoA production and de novo lipogenesis, supplying essential building blocks for proliferating tumour cells.^45^ This broad regulatory scope enables O‐GlcNAcylation to integrate glucose, amino acid and lipid metabolism, establishing it as a master coordinator of tumour metabolism beyond its role as a stress sensor.

In summary, elevated O‑GlcNAcylation levels are a common feature in many cancers, promoting tumour cell growth and survival through modulation of signalling pathways and metabolic networks. Given its central role in tumour signalling and metabolic remodelling, developing specific inhibitors (e.g., the OGT inhibitor OSMI‑1) or combination therapies targeting this modification holds significant clinical potential.

## 4 AUTOPHAGY: THE ‘SCAVENGER’ OF CELLS

**FIGURE 2:**
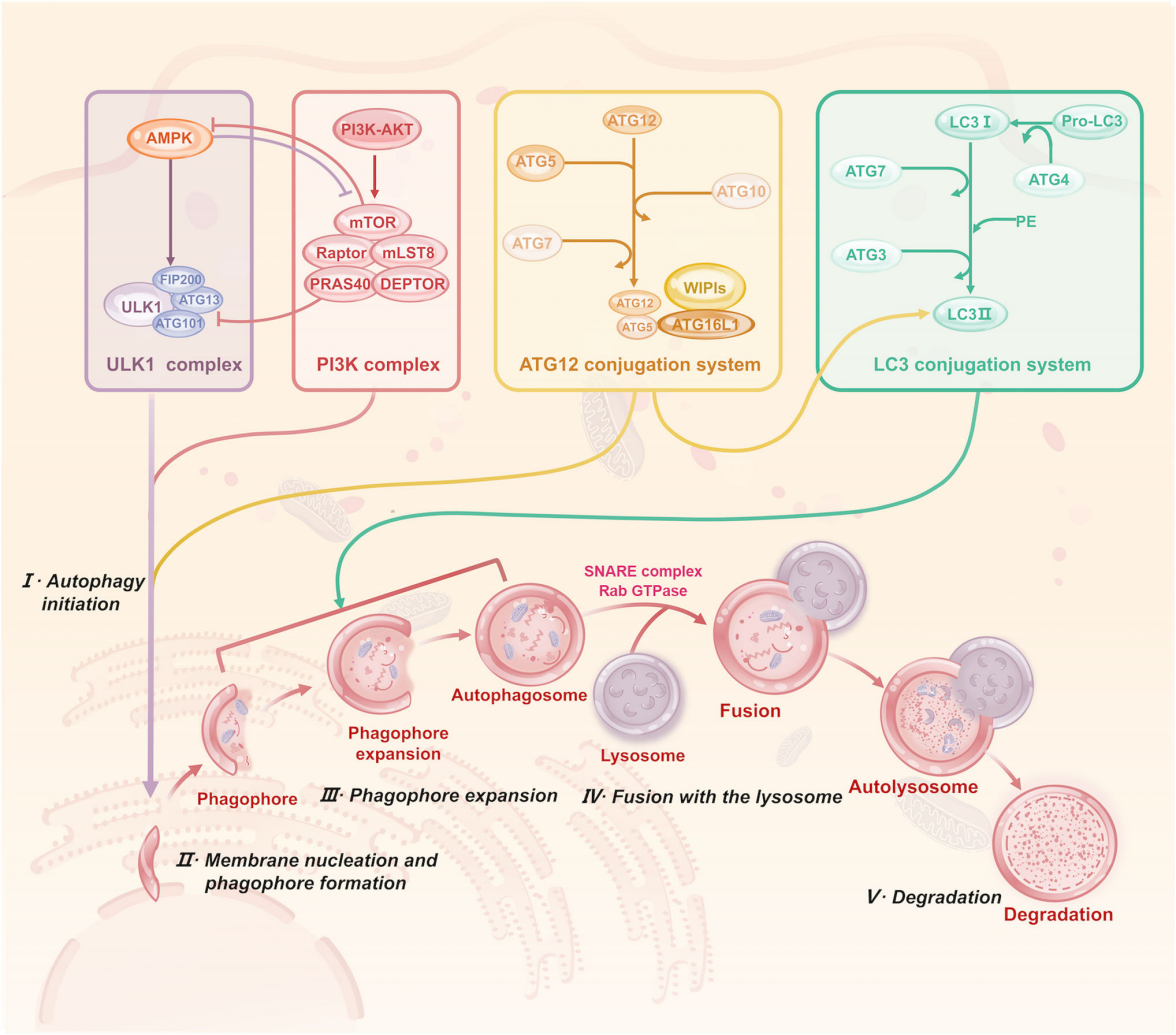
The process and main regulatory mechanisms of autophagy. Autophagy is a tightly regulated degradation process that proceeds through initiation, nucleation, elongation, fusion and degradation. The process begins with the activation of the ULK1 complex (ULK1, FIP200, ATG13, ATG101), which is positively regulated by AMPK under energy stress and inhibited by mTORC1 (mTOR, Raptor, mLST8) via the PI3K/AKT pathway under nutrient‐rich conditions. Activated ULK1 phosphorylates the class III PI3K complex, leading to phosphatidylinositol 3‐phosphate (PI3P) production and phagophore nucleation. Phagophore expansion is driven by two ubiquitin‐like conjugation systems: the ATG12 system (ATG7, ATG10, ATG5, ATG16L1) and the LC3 system (LC3/ATG8, ATG4, ATG7, ATG3), where LC3 is lipidated to LC3‐II with the help of the ATG12–ATG5–ATG16L1 complex. The expanding phagophore engulfs cytoplasmic cargo and seals to form an autophagosome, which then fuses with a lysosome in a process mediated by factors such as SNARE complexes and Rab GTPases to form an autolysosome. Finally, the contents are degraded via lysosomal hydrolases for cellular recycling.

Autophagy, as a conserved self‐defence program in eukaryotic cells, selectively removes misfolded proteins, damaged organelles and pathogens through a lysosome‐dependent degradation system and recycles catabolic products to the cytoplasm to participate in biosynthesis and energy metabolism. This process is governed by a multi‐layer signalling network and can be divided into three major stages (Figure 2). The first is autophagy initiation and isolation membrane formation. Autophagy activation begins with assembling the UNC‐51‐like kinase 1 (ULK1) complex (ULK1–ATG13–ATG101–FIP200). Upon mTORC1 inhibition or AMPK activation, ULK1 is deregulated by dephosphorylation, which in turn phosphorylates the PI3K class CIII complex (VPS34–AMBRA1–Beclin1) and drives endoplasmic reticulum‐derived isolation membrane nucleation.^46^, ^47^ The second step is the maturation of autophagosomes. After autophagosome formation, the PI3K CIII complex produces PI3P that binds to WIPI1 and WIPI2, promoting autophagosome membrane extension. This step relies mainly on two ubiquitin‐like conjugation systems. One is the ATG5–ATG12 system: ATG12 is activated by the E1‐like enzyme ATG7, transferred to the E2‐like enzyme ATG10 and conjugated to ATG5; the ATG12–ATG5 conjugate then recruits ATG16L1 to form an E3‐like complex that facilitates membrane elongation. The second is the microtubule‐associated protein 1A/1B‐light chain 3 (LC3) lipidation system: the protease ATG4 cleaves pro‑LC3 to generate LC3‐I, which is then activated by ATG7 (E1), transferred to ATG3 (E2) and finally conjugated to phosphatidylethanolamine (PE) by the ATG12–ATG5–ATG16L1 complex (E3) to form membrane‑anchored LC3‑II, a hallmark of mature autophagosomes.^48^ The final stage entails autophagosome–lysosome fusion and cargo degradation. Mature autophagosomes are transported along microtubules and fuse with lysosomes via mechanisms mediated by Rab GTPases and soluble‐ethylmaleimide‐sensitive factor attachment protein receptors (SNARE) complexes.^49^, ^50^ Two major SNARE complexes are involved: STX17–SNAP29–VAMP8 mediates canonical fusion, whereas YKT6–SNAP29–STX7 participates in fusion with specific late endosomal/lysosomal compartments.^51^, ^52^ Fusion results in autolysosome formation, where lysosomal hydrolases degrade engulfed contents, and released metabolites are recycled.

Autophagy exhibits a time‐dependent dual function in tumourigenesis and development. In the precancerous stage, autophagy inhibits ROS accumulation and genomic instability by removing damaged mitochondria and misfolded proteins, thereby blocking oncogenic signalling. In progressive tumours, autophagy maintains energetic homeostasis by recycling metabolic substrates and helps cells adapt to stressful microenvironments such as hypoxia and nutrient deprivation, promoting tumour survival and distal metastasis. However, in certain contexts (e.g., specific tumour types or therapy phases), excessive autophagy inhibition may impair tumour cell viability or sensitise cells to alternative death pathways, highlighting context‐dependent outcomes. The interaction between autophagy and apoptosis determines anticancer therapy sensitivity. In protective autophagy, chemotherapy or radiotherapy induced stress can activate autophagy and inhibit cell death by degrading pro‐apoptotic proteins, whereas, in lethal autophagy, over‐activated autophagy can lead to permeabilisation of lysosomal membranes, which releases histone proteases to induce caspase‐independent death. This intricate ‘double‐edged sword’ nature of autophagy is precisely modulated by various PTMs, including O‐GlcNAcylation, which can tip the balance towards tumour suppression or promotion depending on cellular context. Therefore, targeting the autophagy regulatory network becomes an emerging strategy to optimise anticancer therapy, though potential toxicity to normal tissues from systemic autophagy modulation requires careful evaluation.

## 5 REGULATORY MECHANISMS OF O‐GLCNACYLATION ON AUTOPHAGY IN CANCER

To systematically illustrate the key nodes and functional consequences of O‐GlcNAcylation in autophagy regulation across different cancer contexts, we have summarised known O‐GlcNAcylation sites on core autophagy‐related proteins in Table 1. This table provides a concise reference for the following detailed mechanistic discussions.

**TABLE 1:** Summary of O‐GlcNAcylation sites on key autophagy‐related proteins and their functional consequences in cancer.

Protein	O‐GlcNAc site (s)	Functional consequence	Cancer context/model	References
Raptor	Thr700	Promotes interaction with Rag GTPases, enhances lysosomal recruitment and activation of mTORC1, thereby suppressing autophagy initiation	General glucose signalling mechanism	^58^
AKT	Not specified (promotes Ser505 phosphorylation)	Enhances AKT kinase activity, suppressing autophagy	Drosophila melanogaster	^21^
AMPK	Not specified (inhibits Thr172 phosphorylation)	Inhibits kinase activity via steric hindrance; leads to aberrant mTORC1 activation and suppresses autophagy	Colon cancer (LoVo cells), bladder cancer	^64^, ^65^
ULK1	Thr754	Promotes autophagy initiation; enhances ULK1‐mediated Beclin‐1 phosphorylation (Ser15) and ATG14L binding, activating VPS34 complex	Liver, general autophagy under glucose starvation	^73^, ^74^
ULK1	Ser409	Antagonises PKCα‐mediated Ser423 phosphorylation; blocks chaperone‐mediated degradation, stabilises ULK1; enhances STX17 binding and promotes autophagosome–lysosome fusion	HPV+ HNSCC cells	^75^
ATG4B	Not specified	Enhances its proteolytic (hydroxylase) activity towards LC3, promoting LC3 lipidation and autophagosome formation	Neuronal SH‐SY5Y cells (model for metabolic stress)	^80^
ATG7	Not specified	Potential impact on ATG12/LC3 conjugation; regulates autophagosome maturation (evidence from model organisms)	Mouse brain extracts	^21^
LC3‐I	Not specified	Potential impact on lipidation; regulates autophagosome maturation (evidence from model organisms)	Mouse brain extracts	^21^
SNAP‐29	Ser2, Ser61, Thr130, Ser153	Inhibits binding to STX17 and VAMP8; impairs SNARE complex assembly and autophagosome–lysosome fusion	Bladder cancer, ovarian cancer, cervical cancer (HeLa)	^88^, ^89^, ^90^, ^91^
GRASP55	Not specified	Deglycosylation under low glucose promotes its binding to LC3‐II/LAMP2 and activates PI3K–UVRAG complex formation, thereby promoting autophagosome–lysosome fusion.	Breast cancer, general autophagy under nutrient deprivation	^93^

### 5.1 O‐GlcNAcylation modulates autophagosome formation

#### 5.1.1 mTOR‐related signalling pathways

mTOR is a 289‐kDa serine/threonine kinase that operates through two distinct complexes, mTORC1 and mTORC2.^53^ mTORC1 serves as the central regulator of autophagy, with its activity controlled by multiple signalling inputs. Growth factors suppress autophagy initiation by activating the PI3K/AKT and MAPK pathways, which promote mTORC1‐mediated phosphorylation of downstream targets.^54^, ^55^ Conversely, under energetic stress, AMPK phosphorylates Raptor (an mTORC1 subunit), alleviating mTORC1‐mediated autophagy suppression and driving autophagosome formation.^56^, ^57^ Mechanistically, mTORC1 inhibits ULK1 kinase activity via phosphorylation at Ser757; upon mTORC1 inactivation, the ULK1 complex activates, promoting assembly of the Atg12–Atg5–Atg16L1 complex, LC3 lipidation and autophagosome biogenesis.^57^

**FIGURE 3:**
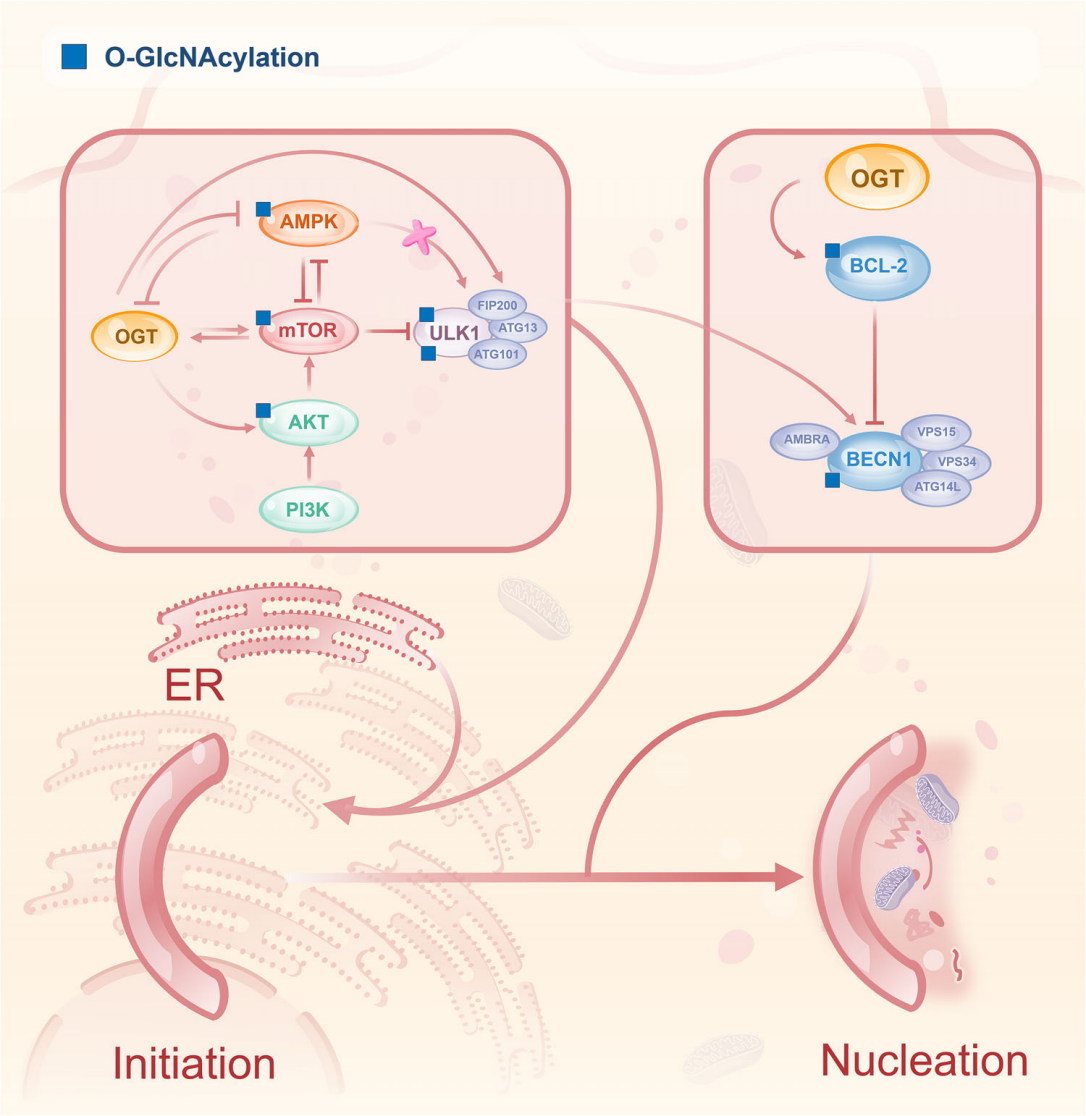
O‐GlcNAcylation regulates autophagy initiation and phagophore formation. This figure illustrates the key regulatory nodes of O‐GlcNAcylation during the autophagy initiation stage. OGT‐mediated O‐GlcNAcylation can directly or indirectly modulate core kinases and scaffold proteins within the pathway. A mutual inhibition exists between OGT/O‐GlcNAcylation and AMPK, whereas a mutual promotion occurs between OGT/O‐GlcNAcylation and mTOR, together forming a fine‐tuned nutrient‐sensing network. Elevated OGT/O‐GlcNAcylation levels can activate mTOR by inhibiting AMPK activity, activating Akt or directly stimulating mTOR, thereby suppressing ULK1 complex activation and inhibiting autophagy initiation. ULK1 itself is also a target of O‐GlcNAcylation. O‐GlcNAcylation of ULK1 stabilises/activates ULK1 or enhances ULK1‐mediated phosphorylation of BECN1 at Ser15, thereby promoting autophagy initiation. Additionally, O‐GlcNAcylation of BCL‐2 promotes its interaction with BECN1, leading to inhibition of autophagy initiation. Arrows indicate activation or promotion; bar‐headed lines (‘T’ shape) represent inhibition or blockade. Key O‐GlcNAcylation sites shown include: AMPK (not specified), Raptor (Thr700), AKT (not specified), ULK1 (Thr754, Ser409), BECN1 (not specified) and BCL‐2 (not specified).

As a metabolism‐sensitive PTM, O‐GlcNAcylation regulates autophagy by targeting the mTOR signalling network at multiple levels (Figure 3). Recent studies have identified a direct mechanism by which O‐GlcNAcylation activates mTORC1: the core component Raptor is glycosylated at Thr700 under glucose‐sufficient conditions. This modification promotes the interaction between Raptor and Rag GTPases, facilitating the lysosomal recruitment and subsequent activation of mTORC1.^58^ This pathway operates alongside the established FBP‐aldolase mechanism for glucose sensing. mTORC1 activity is also negatively regulated by AMPK, which phosphorylates Raptor to inhibit its function – a key counter‑regulatory mechanism (detailed in Section 5.1.2). In the canonical PI3K/AKT/mTOR pathway, AKT phosphorylates mTOR at Ser1448 to enhance its pro‐proliferative function. Drosophila studies demonstrate that O‐GlcNAcylation increases AKT phosphorylation at Ser505, enhancing its kinase activity and suppressing autophagy.^21^ Similarly, elevated O‐GlcNAcylation in cervical cancer activates the IGF1R/PI3K/AKT pathway, promoting proliferation, migration and chemoresistance.^59^ Thus, O‐GlcNAcylation can promote mTORC1 signalling through both direct glycosylation of its core component and potentiation of upstream activators.

O‐GlcNAcylation and mTOR exhibit bidirectional, tissue‐specific regulation (Figure 3). In colon cancer, GFAT inhibitors reduce O‐GlcNAcylation, suppressing mTOR activation, while OGA knockdown (KD) elevates O‐GlcNAcylation, enhancing mTOR signalling and tumour progression.^60^ Conversely, mTOR inhibitors in hepatocellular carcinoma decrease global O‐GlcNAcylation by promoting OGT degradation via ubiquitin–proteasome and selective autophagy pathways, establishing a negative feedback loop.^61^ In breast cancer, mTOR activation up‐regulates OGT expression through a c‐MYC‐independent mechanism, exacerbating metabolic reprogramming via elevated O‐GlcNAcylation.^62^ Thus, O‐GlcNAcylation acts both upstream and downstream of mTOR, forming adaptive loops that reinforce pro‐tumourigenic signalling and suppress autophagy in nutrient‐rich conditions. The direct glycosylation of Raptor represents a precise mechanism by which the nutrient sensor O‐GlcNAc fine‐tunes the central autophagy regulator mTORC1 in response to glucose availability.^58^ This dynamic interplay advances our understanding of tumour metabolic adaptation. It informs novel therapeutic strategies targeting the mTOR–O‐GlcNAcylation axis.

#### 5.1.2 AMPK‐related signalling pathways

The AMPK pathway – a core metabolic regulator independent of mTOR – orchestrates energy stress responses and autophagy activation by sensing intracellular AMP/ATP dynamics. During energy deprivation, AMPK induces autophagy through dual mechanisms: (1) indirect regulation of mTOR activity and (2) direct phosphorylation and activation of ULK1 at multiple sites including Ser317 and Ser777 to initiate autophagosome formation.^63^ A primary mechanism for AMPK to inhibit mTORC1 is through direct phosphorylation of its subunit Raptor. Phosphorylation of Raptor at Ser792 not only directly inhibits mTORC1 activity but also antagonises the glucose‐induced O‐GlcNAcylation of Raptor at Thr700, thereby disrupting the Raptor–Rag interaction and lysosomal recruitment of mTORC1.^58^ This creates a reciprocal switch wherein O‑GlcNAcylation and phosphorylation compete to regulate mTORC1 in accordance with nutrient and energy levels. AMPK further fine‐tunes autophagic flux and metabolic homeostasis by modulating downstream effectors (e.g., TSC1/2‐Rheb complex).

O‐GlcNAcylation and AMPK engage in a bidirectional ‘molecular balance’ for metabolic sensing (Figure 3). In bladder cancer, O‐GlcNAcylation of the AMPKα subunit inhibits its kinase activity via steric hindrance, inactivating ULK1, suppressing autophagy and promoting tumour survival.^64^ Furthermore, in colon cancer models, O‐GlcNAcylation of AMPK itself inhibits its phosphorylation at the critical activation loop (Thr172), leading to aberrant mTORC1 activation and suppressed autophagy.^65^ Conversely, AMPK activation in vascular endothelial cells reduces O‐GlcNAcylation by inhibiting GFAT1 (the HBP rate‐limiting enzyme), augmenting VEGF‐mediated angiogenesis.^66^ Intriguingly, AMPK and OGT form a regulatory loop: AMPK phosphorylation alters OGT's subcellular localisation and substrate specificity. At the same time, OGT‐mediated AMPK glycosylation modulates its phosphorylation status.^67^, ^68^ This reciprocal inhibition creates a metabolic switch: nutrient sufficiency favours O‐GlcNAcylation and mTOR‐driven anabolism, while energy stress activates AMPK to suppress O‐GlcNAcylation and induce catabolic autophagy. This crosstalk is pivotal in tumour metabolic reprogramming, and delineating the AMPK–O‐GlcNAcylation network may yield novel anticancer therapies targeting autophagy‐metabolism integration.

#### 5.1.3 O‐GlcNAcylation targets the ULK1 complex to regulate autophagy initiation

The ULK1 complex acts as a central autophagy initiation hub in mammalian cells, integrating nutrient and stress signals.^69^ ULK1, a serine/threonine kinase, critically regulates autophagy induction via PTMs.^70^ Under nutrient‐replete conditions, mTORC1 phosphorylates ULK1 to inhibit its kinase activity and block autophagy. During energy stress, mTORC1 dissociates from ULK1, enabling ULK1 translocation to ER‐derived isolation membranes to trigger autophagosome nucleation.^57^, ^71^ AMPK further activates ULK1 through direct phosphorylation at Ser317 and Ser777, creating a dual energy‐sensing switch.^72^

Recent studies identify ULK1/2 as a key node for O‐GlcNAcylation‐mediated autophagy regulation (Figure 3). Glucose deprivation elevates OGT‐dependent O‐GlcNAcylation at ULK1 Thr754, which occurs downstream of AMPK phosphorylation, forming a sequential regulatory cascade.^73^, ^74^ ULK1 glycosylation modulates autophagy through multiple mechanisms: it enhances ULK1‐mediated Beclin‐1 phosphorylation at Ser15 and promotes ATG14L binding, activating the VPS34 lipid kinase complex to drive autophagosome formation.^73^, ^74^ Additionally, dephosphorylation of ULK1 Ser757 removes steric constraints, facilitating O‐GlcNAcylation and amplifying autophagy signalling via positive feedback.^74^ In head and neck squamous carcinoma (HNSCC), HPV‐induced O‐GlcNAcylation at ULK1 Ser409 antagonises PKCα‐mediated Ser423 phosphorylation, blocks chaperone‐mediated degradation, stabilises ULK1, enhances STX17 binding and ultimately promotes autophagosome–lysosome fusion.^75^ The context‐dependent outcomes of ULK1 O‐GlcNAcylation – promoting initiation under glucose starvation (Thr754) or enhancing flux under oncogenic HPV signalling (Ser409) – exemplify how this modification can tune the ‘double‐edged sword’ of autophagy to favour tumour cell survival under diverse stresses. These insights expand the autophagy regulatory paradigm and highlight ULK1 glycosylation as a therapeutic target for tumour metabolism modulation.

### 5.2 O‐GlcNAcylation regulates autophagosome maturation

After forming pre‐autophagosomes, the autophagosome membrane elongates until it completely engulfs the cargo. Two ubiquitinated protein modification systems primarily mediate this process. The first system involves the ATG5–ATG12 complex. ATG12 binds to ATG5 with the assistance of E1‐like ubiquitin activase ATG7 and E2‐like ubiquitin transferase ATG10, forming the ATG12–ATG5 complex. This complex associates with ATG16 to generate the ATG12–ATG5–ATG16 complex, which exhibits E3‐like ubiquitin ligase activity.^76^, ^77^ The second system concerns LC3 processing. LC3 is cleaved by the cysteine protease ATG4 to produce cytoplasmic soluble LC3‐I, which is activated by ATG7 and then covalently bound to PE to generate lipid‐soluble LC3‐PE and LC3‐II under the interaction of ATG3 and ATG12–ATG5–ATG16 complexes.^78^, ^79^ LC3‑II stably decorates both the inner and outer autophagosomal membranes until fusion with lysosomes, serving as a key marker of autophagosome maturation.

**FIGURE 4:**
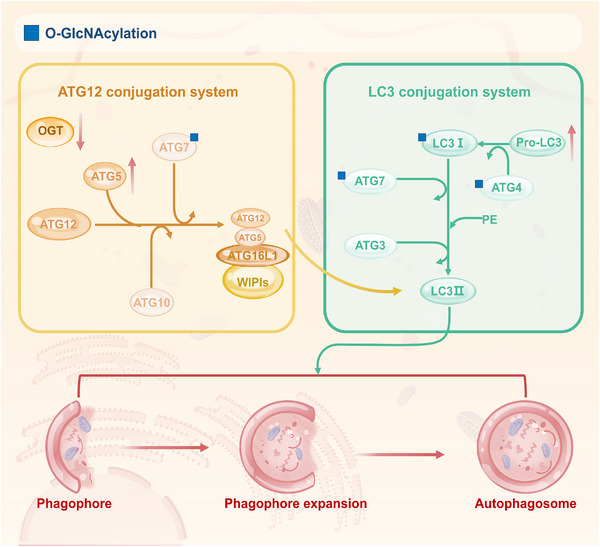
O‑GlcNAcylation regulates phagophore expansion and autophagosome maturation by modulating the ATG12/LC3 conjugation systems. This figure illustrates the regulatory role of O‑GlcNAcylation in the two ubiquitin‑like conjugation systems during autophagosome formation. Down‐regulation of OGT significantly increases the protein levels of ATG5, the ATG5–ATG12 complex and pro‑LC3, suggesting that O‑GlcNAcylation may negatively regulate the stability or processing of these proteins. ATG4, LC3‑I and ATG7 have been identified as molecular targets of O‑GlcNAcylation. O‑GlcNAcylation of ATG4 enhances its protease activity, thereby promoting the lipidation of LC3‑I to LC3‑II and positively regulating autophagosome formation. O‑GlcNAcylation of LC3‑I and ATG7 may influence the efficiency of autophagosomal membrane expansion. These findings reveal that O‑GlcNAcylation finely tunes autophagosome maturation through multiple targets. In the figure, ‘↑’ indicates up‑regulation of protein expression or activity and ‘↓’ indicates down‑regulation.

Several in model organisms indicate that O‐GlcNAcylation dynamically regulates autophagosome maturation (Figure 4). In Drosophila larvae, OGT down‐regulation significantly increases protein levels of ATG8 and ATG5, even in the absence of starvation.^21^ Recent work in neuronal SH‐SY5Y cells demonstrated that ATG4B, the key protease responsible for LC3 processing, is a direct target of O‐GlcNAcylation. Under metabolic stress (e.g., low glucose) or upon pharmacological elevation of O‐GlcNAc levels (via OGA inhibition), ATG4B O‐GlcNAcylation enhances its proteolytic activity towards LC3, thereby promoting LC3 lipidation and autophagosome formation.^80^ In addition, in mouse brain extracts, it has been observed that ATG7 and LC3‐I can undergo O‐GlcNAcylation, potentially modulating their activity or stability and thereby impacting autophagy signalling in mammals.^21^ In another study conducted on C. elegans, researchers observed elevated that mutants deficient in O‐GlcNAc cycling enzymes show elevated levels of GFP::LGG‑1 (an ATG8/LC3 homolog) and its PE‑modified form upon starvation^81^ (Figure 4). These cross‐species findings suggest that O‐GlcNAcylation can negatively regulate key components of the autophagosome maturation machinery, with ATG4B representing a newly identified positive regulatory node whose modification enhances autophagic flux under stress. The precise sites and mechanistic consequences of O‐GlcNAcylation on ATG5, ATG7 and LC3 require further mapping in mammalian cancer models.

### 5.3 O‐GlcNAcylation modulates autophagosome–lysosome fusion

After autophagosomes complete maturation, they are driven by dynamin for targeted transport along the microtubule system to the lysosomal region, where they form autophagic lysosomes through membrane fusion. Lysosomal acid hydrolases degrade autophagosome‐coated substrates and release breakdown products into the cytoplasm to participate in metabolic cycles. Autophagosome–lysosome fusion is a central event in the terminal phase of autophagy. Its precise regulation relies on the synergistic action of multiple membrane dynamics factors, including SNARE complexes, tethering proteins and Rab GTPase family members.^82^ Key regulators include: (1) SNARE complexes: STX17–SNAP‐29–VAMP8 and YKT6–SNAP‐29–STX7 mediate specific fusion with lysosomal/late endosomal subtypes.^51^, ^52^ (2) Tethering complexes: The homotypic fusion and protein sorting (HOPS) complex facilitates docking and enhances fusion efficiency.^83^, ^84^ (3) Rab GTPases: Rab7 is crucial for recruiting effectors like HOPS and coordinating late endosomal/lysosomal motility and fusion.^83^, ^84^ (4) Lysosomal membrane proteins: LAMP1 and LAMP2 stabilise lysosomal membranes and interact with fusion machinery components.^85^, ^86^, ^87^ (5) Golgi protein GRASP55: Acts non‐canonically by bridging LC3‐II to LAMP2 and promoting PI3K complex assembly via Beclin‐1/UVRAG recruitment.^85^, ^86^, ^87^

**FIGURE 5:**
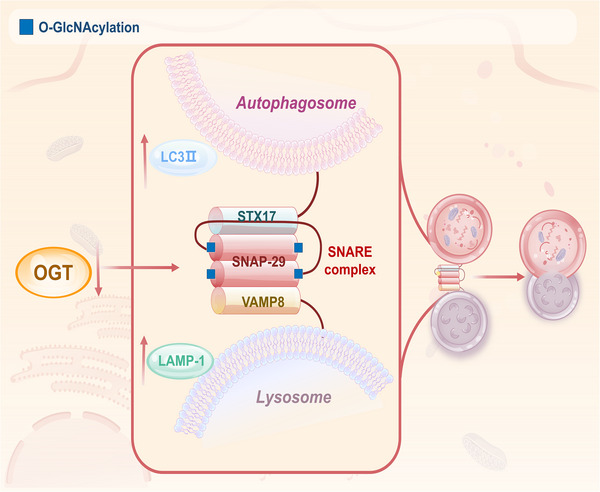
O‐GlcNAcylation regulates autophagosome and lysosome fusion. This figure illustrates the key inhibitory role of O‑GlcNAcylation during autophagosome–lysosome fusion. SNAP‑29, a component of the SNARE complex, is a major target of O‑GlcNAcylation, primarily modified at sites including Ser2, Ser61, Thr130 and Ser153. Reduction in O‑GlcNAcylation of SNAP‑29 enhances its binding affinity for STX17 and VAMP8, thereby promoting the fusion process. Moreover, down‐regulation of OGT concomitantly up‐regulates the expression of the lysosomal marker LAMP‑1 and the autophagosomal marker LC3‑II, suggesting that O‑GlcNAcylation may coordinately regulate fusion efficiency through multiple targets. In the figure, ‘↑’ indicates up‑regulation of protein expression or activity and ‘↓’ indicates down‑regulation.

As the molecular ‘rivet’ of autophagosome–lysosome fusion, the SNARE complex is dynamically regulated by O‐GlcNAcylation. Although the SNARE complex consists of multiple proteins, only SNAP‐29 has been shown to interact with OGT and undergo O‐GlcNAcylation, with major glycosylation sites at Ser2, Ser61, Thr130 and Ser153.^88^ Reducing the O‐GlcNAcylation level of SNAP‐29 can enhance its binding strength to STX17 and VAMP8, accelerating the fusion process and enhancing the autophagy flux.^89^, ^90^ This mechanism is relevant in toxicological and anticancer contexts. For instance, environmental arsenic inhibits autophagic flux at low concentrations by enhancing SNAP‐29 O‐GlcNAcylation, thereby disrupting SNARE complex assembly.^91^ Conversely, the small molecule SM15, a late‐stage autophagy inhibitor, acts by enhancing SNAP‐29 O‐GlcNAcylation to block SNARE complex formation, leading to autophagosome and ROS accumulation and apoptotic cell death.^89^ Furthermore, inhibition of OGT expression synchronously up‐regulated the expression of lysosomal marker protein LAMP‐1 and autophagosome marker LC3‐II, suggesting that O‐GlcNAcylation synergistically regulates the fusion efficiency through multiple targets^92^ (Figure 5). As an energy‐sensing element, the O‐GlcNAcylation status of GRASP55 directly responds to glucose/amino acid deprivation: under low‐glucose conditions, GRASP55 deglycosylates in response to the fusion process. GRASP55 deglycosylation strengthens its binding ability to LC3‐II/LAMP2 and promotes autophagosome–lysosome fusion by activating PI3K–UVRAG complex formation, a dual pathway.^93^ Thus, O‐GlcNAcylation exerts a net inhibitory effect on the final fusion step, primarily through modifications of SNAP‐29 and GRASP55, to fine‐tune autophagic flux in response to nutrient status.

### 5.4 Genetic knockout models validate the specificity of O‐GlcNAcylation in autophagy regulation

Loss‐of‐function studies employing genetic knockout (KO) or knockdown (KD) models in cells and animals are indispensable for delineating the precise, context‐dependent roles of O‐GlcNAcylation within the autophagy cascade in cancer. By ablating the modifying enzymes (OGT/OGA) or introducing point mutations at key glycosylation sites on autophagy‐related proteins, these approaches establish causality and reveal critical regulatory patterns.

*Targeting the ‘writers’ and ‘erasers’*: In cellular models such as bladder cancer, OGT KD consistently up‐regulates autophagic flux, evidenced by increased LC3‐II lipidation and decreased p62/SQSTM1 accumulation.^64^, ^92^ Conversely, OGA KO or inhibition leads to global hyper‐O‐GlcNAcylation, exerting context‐dependent, biphasic effects on autophagy. For instance, in bladder cancer models, OGA inhibition may suppress autophagy by excessively modifying nodes like AMPK,^64^ whereas in other contexts, OGA mutants can paradoxically induce autophagy.^81^ These opposing results highlight the dual role of autophagy in cancer, wherein genetic perturbation of O‐GlcNAc cycling can shift the balance towards either cell survival or death, depending on cellular context. Collectively, these data indicate that the interplay between OGT and OGA activities finely tunes autophagic flux in a tumour microenvironment‐dependent manner.

*Dissecting specific autophagy regulators*: CRISPR/Cas9‑mediated KO of ULK1 abolishes the stimulatory effect of glucose starvation on autophagy initiation, confirming its essential role in nutrient sensing via O‑GlcNAcylation.^73^, ^74^ Mutating the identified O‐GlcNAc site on ULK1 (Thr754) disrupts its interaction with the ATG14L–VPS34 complex, providing residue‐level mechanistic evidence.^74^ In HPV‐positive HNSCC models, O‐GlcNAcylation at ULK1 Ser409 antagonises PKCα‐mediated phosphorylation at Ser423, blocks ULK1 degradation, stabilises the protein and promotes autophagic flux.^75^ This demonstrates that O‐GlcNAcylation at different sites on the same protein can regulate autophagy through distinct mechanisms. Genetic evidence extends to later stages of autophagy. For example, point mutations at O‐GlcNAcylation sites on ATG4B compromise its proteolytic activity towards LC3 and attenuate stress‐induced autophagosome formation, directly validating its regulatory function in autophagosome maturation.^80^ Similarly, studies employing glycosylation‐defective GRASP55 mutants demonstrate that its nutrient‐sensitive O‐GlcNAcylation is essential for promoting autophagosome–lysosome fusion under metabolic stress.^93^ For the fusion step, siRNA‑mediated depletion of SNAP‐29 impairs fusion in models such as ovarian cancer.^90^ Rescue experiments using glycosylation‐deficient SNAP‐29 mutants demonstrate that O‐GlcNAcylation at specific residues (e.g., Ser2, Thr130) directly modulates SNARE complex assembly and fusion efficiency.^88^ Definitive genetic evidence is provided by studies in specific cancer models: re‐expression of an O‐GlcNAcylation‐defective SNAP‐29 mutant, but not wild‐type SNAP‐29, rescues the fusion block induced by arsenic in cervical cancer HeLa cells or by the cytotoxic compound SM15 in treated cancer cells.^89^, ^91^

*Insights from in vivo models*: The in vivo relevance is supported by studies in tissue‐specific KO mice. For example, liver‐specific OGT deletion perturbs metabolic homeostasis and alters the expression of core autophagy proteins, linking systemic O‐GlcNAc flux to stress‐adaptive autophagy in a setting relevant to hepatocellular carcinoma.^73^ These in vivo findings critically connect molecular mechanisms to physiological and pathophysiological outcomes, highlighting the translational relevance of this regulatory axis.

In summary, genetic KO and targeted mutagenesis studies provide a high‐resolution map of the O‐GlcNAcylation‐autophagy network. They establish causality and reveal the precise molecular nodes where O‐GlcNAcylation fine‐tunes autophagy in a cancer‐type and context‐dependent manner. The precision of modern gene‐editing tools, such as CRISPR/Cas9‐based base editing, now enables the systematic functional mapping of specific glycosylation sites within the autophagy machinery, offering unprecedented clarity on its crosstalk with other PTMs. This work validates the specificity of this PTM, highlights its therapeutic potential and emphasises that future mechanistic explorations must account for tumour heterogeneity. Integrating these genetically validated mechanisms with patient‐relevant models is a crucial next step for developing stratified therapeutic interventions.

### 5.5 A context‐dependent summary model of O‐GlcNAcylation in autophagy regulation

The regulatory impact of O‐GlcNAcylation on autophagy is not uniform but exhibits significant context‐dependence, influenced by tumour type, nutrient status, specific protein targets and modification sites. To synthesise these complex interactions, we present a summary model (Table 2) organising the dual and sometimes opposing effects of O‐GlcNAcylation across different contexts. This model underscores its role as a tunable ‘molecular switch’ that can either promote or suppress autophagic flux to favour tumour cell adaptation.

**TABLE 2:** Context‐dependent effects of O‐GlcNAcylation on autophagy in cancer.

Cancer context/model	Target/site (s)	Effect on autophagy	Proposed outcome for tumour	References
Nutrient sufficiency/various cancers	AMPK (inhibition)	Suppresses initiation	Promotes anabolic growth	^64^, ^65^
	AKT/mTOR (activation)	Suppresses initiation	Promotes proliferation and survival	^21^, ^59^
Glucose sufficiency/general signalling	Raptor (Thr700)	Suppresses initiation	Promotes anabolic growth and proliferation via sustained mTORC1 activity	^58^
Glucose starvation/liver cancer	ULK1 (Thr754)	Promotes initiation	Enhances stress adaptation	^73^, ^74^
HPV infection/HNSCC	ULK1 (Ser409)	Promotes fusion	Stabilises ULK1, enhances flux	^75^
Metabolic stress/neuronal models	ATG4B	Promotes maturation	Enhances LC3 processing and flux	^80^
Arsenic exposure/cervical cancer	SNAP‐29 (hyper‐O‐GlcNAc)	Inhibits fusion	Blocks flux, induces dysfunction	^91^
Therapeutic intervention (e.g., SM15)	SNAP‐29 (hyper‐O‐GlcNAc)	Inhibits fusion	Induces lethal autophagy/apoptosis	^89^
Low glucose/breast cancer	GRASP55 (deglycosylation)	Promotes fusion	Activates PI3K–UVRAG, enhances flux	^93^
OGA inhibition/bladder cancer	AMPK (hyper‐O‐GlcNAc)	Suppresses initiation	May promote survival	^64^
OGT knockdown/various models	Global reduction	Generally promotes flux	Sensitises to stress or therapy	^64^, ^92^

## 6 CONCLUSIONS

O‐GlcNAcylation acts as a dynamic regulatory hub for nutrient metabolism and the stress response in eukaryotic cells, precisely coordinating cellular signalling networks and protein homeostasis by sensing fluctuations in intracellular glucose, amino acid and energy levels. Aberrant levels of this modification can reshape the transcriptomic and translational landscapes, driving malignant phenotypes including drug resistance and metastasis.^37^, ^94^, ^95^ Autophagy, a central mechanism for intracellular recycling, is dynamically regulated by O‐GlcNAcylation to sustain metabolic adaptation within the tumour stress microenvironment. Targeted intervention in this regulatory axis offers a novel molecular perspective for deciphering the stress survival strategy of tumour cells.

Based on the synergistic role of O‐GlcNAcylation and autophagy in tumourigenesis, the development of therapeutic strategies that co‐target this modification on autophagy‐related proteins holds significant clinical promise. Potential directions include: (i) modulating ULK1/VPS34 complex activity to regulate autophagy initiation and phagophore nucleation; (ii) targeting the LC3 lipidation system (e.g., ATG4/ATG7) to control autophagosome maturation; and (iii) fine‐tuning autophagosome–lysosome fusion efficiency by affecting SNARE complexes (e.g., SNAP‐29/STX17) and associated regulators (e.g., the homotypic fusion and protein sorting (HOPS) complex).

The druggability of the O‐GlcNAc‐autophagy axis is an area of active preclinical investigation, underscored by the development of several small‐molecule inhibitors. Key tool compounds include OGT inhibitors (e.g., OSMI‐1, OSMI‐4) and OGA inhibitors (e.g., Thiamet‐G, MK‐8719), which have provided proof‐of‐concept that pharmacologically modulating O‐GlcNAcylation can alter tumour progression and autophagic flux. For successful clinical translation, however, challenges regarding compound selectivity, pharmacokinetics and potential on‐target toxicity must be addressed.^96^ Critical lessons can be drawn from previous clinical trials of autophagy modulators like hydroxychloroquine in oncology, which highlighted issues of systemic toxicity and the necessity of patient stratification. A promising translational strategy may involve combining lower, better‐tolerated doses of OGT/OGA inhibitors with standard chemotherapy, targeted therapy or immunotherapy. This nuanced approach, guided by monitoring of autophagy status, holds potential to enhance therapeutic efficacy, overcome resistance and minimise toxicity to normal tissues.

Therefore, based on the mechanistic insights discussed, targeting the O‐GlcNAcylation‐autophagy axis represents a strategy with potentially superior molecular specificity and a more favourable safety profile compared with traditional, broad‐spectrum autophagy modulators.

Moving forward, research efforts ought to prioritise several critical areas: first, to systematically analyse the dual biological functions of autophagy at different stages of tumour evolution (primary foci formation, metastatic colonisation and therapeutic resistance), and second, to elucidate the spatial and temporal regulation of novel autophagy regulatory elements (e.g., ATG9 vesicle transport system, ATG14‐mediated PI3K complex assembly) by O‐GlcNAcylation. Beyond this, exploring the potential crosstalk between O‐GlcNAcylation and other nutrient‐sensitive PTMs, such as acetylation and lactylation, in coordinating autophagy flux within the tumour microenvironment represents an exciting frontier for understanding the complex metabolic regulation of cancer. Translational efforts must prioritise the development of highly specific OGT/OGA inhibitors or allosteric modulators and rigorously validate their efficacy – particularly in rational combination regimens – within preclinical models that recapitulate human tumour heterogeneity and therapy resistance. Ultimately, the precise remodelling of tumour autophagy homeostasis via this axis offers an innovative pathway for developing more effective and individualised anticancer therapies.

## AUTHOR CONTRIBUTIONS

Y.Z.L. and L.G. were responsible for the conceptualisation and design of the images as well as the writing of the paper and were the main contributors to the writing of the manuscript. S.M.L. and J.J.Z. were responsible for the collection of the relevant literature. K.Q.Z. was responsible for the initial revision of the article as well as for the review. W.H.R. was responsible for the final review and finalisation of the manuscript for publication.

## ETHICS STATEMENT

The authors have nothing to report.

## CONFLICT OF INTEREST STATEMENT

The authors declare no conflicts of interest.

## Data Availability

Data sharing not applicable to this article as no datasets were generated or analysed during the current study.
